# miR-26a-5p Regulates Adipocyte Differentiation via Directly Targeting *ACSL3* in Adipocytes

**DOI:** 10.1080/21623945.2023.2166345

**Published:** 2023-01-29

**Authors:** Ning Ding, Wenwen Wang, Jun Teng, Yongqing Zeng, Qin Zhang, Licai Dong, Hui Tang

**Affiliations:** a Shandong Provincial Key Laboratory of Animal Biotechnology and Disease Control and Prevention, College of Animal Science & Technology, Shandong Agricultural University, Taian, Shandong Province, China; b Key Laboratory of Efficient Utilization of Non-grain Feed Resources (Co-construction by Ministry and Province), Ministry of Agriculture and Rural Affairs, Shandong Agricultural University, Taian, China; c Shandong Futong Agriculture & Animal Husbandry Development Co. LTD, Linyi, China

**Keywords:** miR-26a-5p, ACSL3, porcine preadipocyte differentiation, adipogenesis

## Abstract

Preadipocytes become mature adipocytes after proliferation and differentiation, and although many genes and microRNAs have been identified in intramuscular fat, their physiological function and regulatory mechanisms remain largely unexplored. miR-26a-5p has been reported to be related to fat deposition, but its effect on porcine preadipocyte differentiation has not been explored. In this study, bioinformatics analysis and luciferase reporter assay identified that miR-26a-5p binds to the 3ʹUTR of Acyl-CoA synthetase long-chain family member 3 (*ACSL3*) mRNA. The model for porcine intramuscular preadipocyte differentiation was established to explore the function of miR-6a-5p-*ACSL3* on adipocyte differentiation. *ACSL3* knockdown markedly reduced the triglycerides (TG) content of cells, as well as the mRNA levels of adipogenic marker genes (*PPAR-γ* and *SREBP-1c*). The number of lipid droplets in cells transfected with a miR-26a-5p mimic is significantly reduced, consistent with *ACSL3* knockdown results, while the miR-26a-5p inhibitor resulted in opposite results. Taken together, miR-26a-5p is a repressor of porcine preadipocyte differentiation and plays a vital role in *ACSL3*-mediated adipogenesis.

## 1. Introduction

Adipose tissue not only stores energy in the form of triglycerides (TG) but also plays an important role in the endocrine system and the regulation of energy balance [1,2]. In pork, intramuscular fat (IMF) content is a primary indicator of meat quality [1,2], and unravelling the genetic mechanisms that affect IMF content will enable the pork industry to improve selective breeding criteria and increase meat value.

Long chain fatty acids (LCFA) generally refer to the fatty acids containing 12–20 C in the carbon skeleton, which are the most abundant in the daily diet of organisms. The syntheses of long-chain ACS (ACSL) family, consisting of ACSL1, ACSL3, ACSL4, ACSL5, and ACSL6 [3], catalyse the formation of long-chain acyl coenzyme A [4,5]. According to the similarity of fatty acid substrate selection and coding sequence, these ACSL family members can be divided into two subfamilies: ACSL3 and ACSL4 belong to one subfamily, and other members are in another subfamily [6–8]. In our previous study, *ACSL3* was identified as a differentially expressed gene between high and low backfat thicknesses of Yimeng black pigs [9]. *ACSL3* is abundantly expressed in endoplasmic reticulum and mitochondria and plays an important role in lipid synthesis, protein modification and β-oxidation [10], and inhibition of intestinal *ACSL3* can reduce lipid synthesis [11].

MicroRNAs have been defined as a kind of endogenous noncoding RNAs, highly conserved in different species [12], that function in RNA silencing by inhibiting or degrading the target gene mRNA after transcription. Many microRNAs have been identified, and their functions have been confirmed in the process of adipogenesis or lipid metabolism. For example, miR-103 and miR-375 can promote the proliferation and differentiation of porcine subcutaneous adipocytes [13,14], while miR-429 and miR-425-5p inhibit the proliferation and differentiation of porcine subcutaneous and intramuscular adipocytes [15,16].

The miR-26 family, which is composed of miR-26a and miR-26b, plays a crucial role in tumorigenesis by targeting critical regulators involved in cell development [17], differentiation [18], and cell cycle [19]. Moreover, miR-26a/b regulates TG synthesis by directly targeting insulin-induced gene 1 (*INSIG1*) [20,21] and is involved in the browning process of adipocytes [22]. miR-26a regulates insulin signalling and glucose and lipid metabolism [23], as well as fatty acid and cholesterol homoeostasis in HepG2 cells to protect against the progression of NAFLD in vitro [24]. These studies suggest that miR-26a may play an important role in lipid droplet synthesis in pigs.

Little is known about the expression pattern and regulation of miR-26a-5p and *ACSL3* in the proliferation and differentiation of IMF cells. In this study, we demonstrated that *ACSL3* plays an important role in the differentiation of porcine adipocyte cells, and miR-26a-5p is a repressor of porcine preadipocyte differentiation and plays a vital role in *ACSL3*-mediated adipogenesis.

## 2. Results

### 2.1 Biological functions of miR-26a-5p based on target analysis

**Figure 1.**
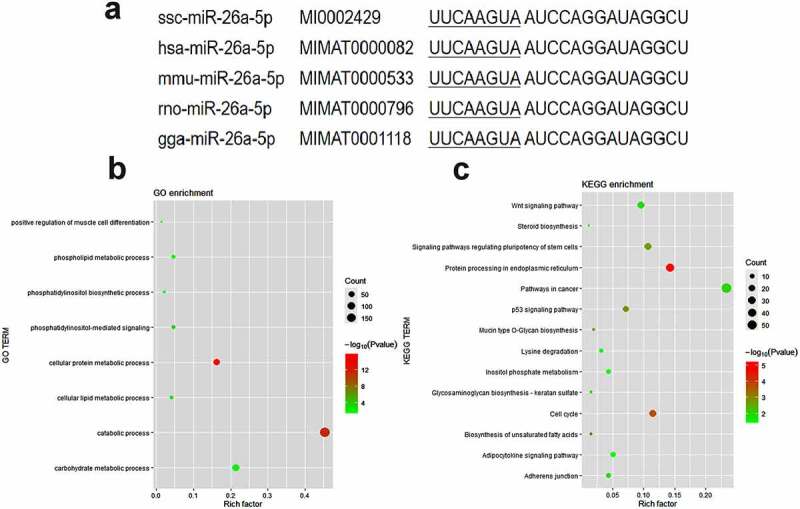
Functional enrichment analysis of predicted miR-26a-5p targets. (a) Mature sequence of miR-26a-5p is conserved among species including pig (ssc), human (hsa), mouse(mmu), rat (rno) and chicken(gga). (b) GO categories of genes targeted by miR-26a-5p. (c) KEGG pathways of genes targeted by miR-26a-5p.

The miR-26a-5p sequences are highly conserved in a variety of species, including pigs, humans, mice, rats, and gorillas (Figure 1(a)). To explore the potential biological roles of miR-26a-5p, we use Gene Ontology (GO) term enrichment and Kyoto Encyclopaedia of Genes and Genomes (KEGG) pathway analyses on miRPathv3 to analyse the targeting genes of miR-26a-5p (Supplementary Table S1). Several biological processes were enriched in the GO analysis (Figure 1(b)), such as the phosphatidylinositol-mediated signalling, the cellular lipid metabolic process, and the positive regulation of muscle cell differentiation. The adipocytokine signalling pathway and the biosynthesis of unsaturated fatty acids were significantly enriched in the KEGG analysis (Figure 1(c)). Three members of the ACSL family (*ACSL1, ACSL3* and *ACSL4*) were targets of miR-26a-5p predicted by microT-CDS and TarBase (Supplementary Table S1), and they were enriched in the adipocytokine signalling pathway.

### 2.2 Tissue-specific expression of ACSL family members

**Figure 2.**
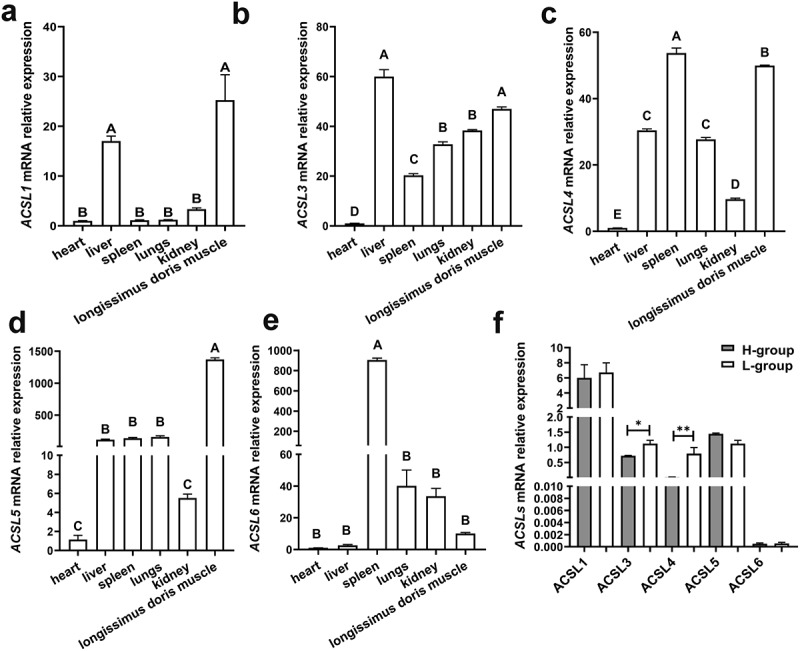
Relative expression levels of *ACSLs*. Tissue specific expression of *ACSL1*(a), *ACSL3* (b), *ACSL4* (c), *ACSL5* (d) and *ACSL6* (e). Relative expression of *ACSLs* in the longissimus dorsi of pigs with higher fat content (H-group) or lower fat content (L-group) (f). Results are presented as means ± SEM; *n* = 3; Labels with different superscripts indicate extremely significantly different values (*P* < 0.01).

The expression of five ACSL members in different tissues of three Yimeng black pigs with high-fat content (H-group) was detected by qPCR. *ACSL1, ACSL3* and *ACSL4* were highly expressed in the liver and longissimus dorsi muscle (Figure 2(a-c)), *ACSL5* was specifically expressed in muscle (Figure 2(d)), and *ACSL6* was specifically expressed in spleen (Figure 2(e)).

We then evaluated the expression levels of *ACSL*1 and *ACSL*3-6 in longissimus dorsi muscle with high-fat content (H-group) and low-fat content (L-group), and found that the expression levels of *ACSL3* and *ACSL4* in the H-group were significantly higher than those in the L-group (Figure 2(f)), hinting they may be important factors in regulating fat content of pigs.

### 2.3 miR-26a-5p Targets the ACSL3 3ʹUTR

**Figure 3.**
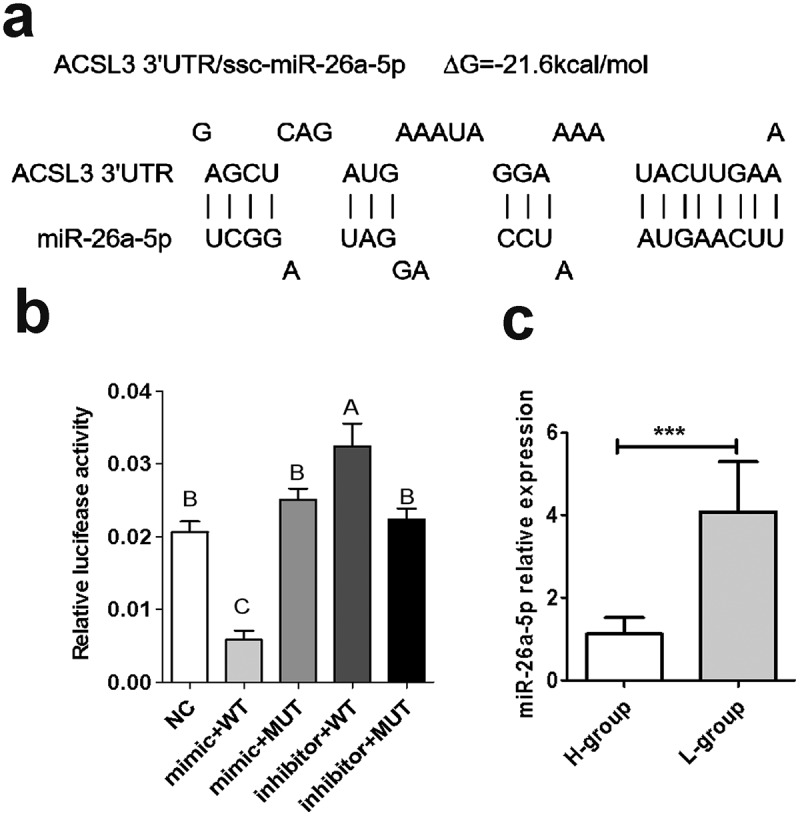
*ACSL3* is a direct target of miR-26a-5p. (a) The predicted interaction between *ACSL3* 3ʹUTR and miR-26a-5p. (b) Dual luciferase reporter assay to detect the interaction of *ACSL3* and miR-26a-5p in PK-15 cells. (c) Relative expression of miR-26a-5p in the longissimus dorsi of pigs with higher fat content (H-group) or lower fat content (L-group). Results are presented as means ± SEM; *n* = 3; *** *P* < 0.001; Labels with different superscripts indicate extremely significantly different values (*P* < 0.01).

*ACSL3* is a potential target gene of miR-26a-5p predicted by microT-CDS, TarBase and TargetScan, the free energy for the interaction between them was predicted to be −21.6 kcal/mol (Figure 3(a)), and the potential miR-26a-5p binding site located within the 3’ untranslated region of *ACSL3* mRNA is shown in Figure 3(a). Dual-luciferase reporter assays revealed that miR-26a-5p mimic significantly decreased the luciferase activity of wild type-ACSL3-plasmid (WT), compared to the negative control. In contrast, miR-26a-5p mimic had no major effect on the mutant-ACSL3-plasmid (MUT) (Figure 3(b)). These data showed that *ACSL3* is a bona fide target of miR-26a-5p. The expression of miR-26a-5p in longissimus dorsi muscle tissues is detected, which revealed a higher expression in L-group than that in H-group (Figure 3c), which is opposite to the expression of *ACSL3*.

### 2.4 ACSL3 promotes adipogenesis differentiation in porcine intramuscular preadipocytes

**Figure 4.**
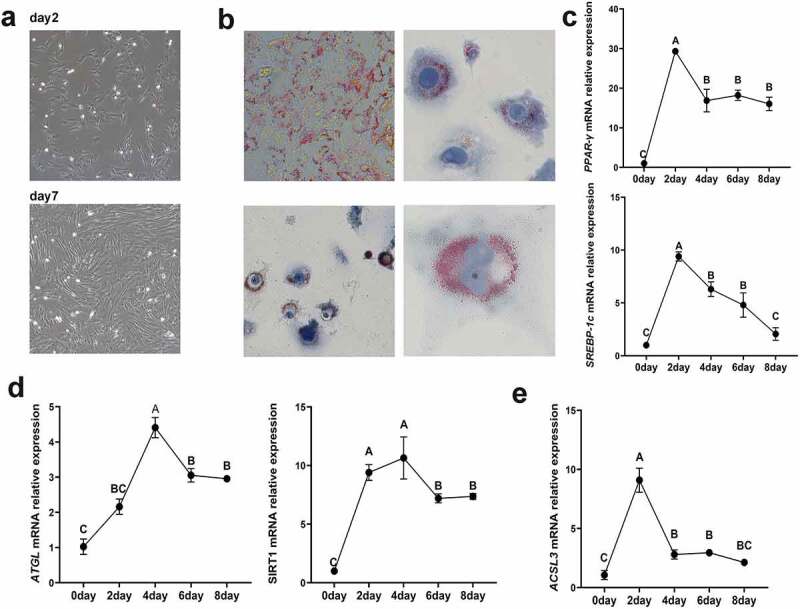
Identification of porcine primary intramuscular preadipocytes. (a) Morphology of primary intramuscular preadipocytes after cultivation for 2 days and 7 days. (b) Lipid accumulation in intramuscular adipocytes after induced differentiation for 4 days. (c) Relative mRNA expression levels of *PPAR-γ* and *SREBP-1c*in cells after induced differentiation for 0, 2, 4, 6 and 8 days. (d) Relative mRNA expression levels of *ATGL* and *SIRT1* in cells after induced differentiation for 0, 2, 4, 6 and 8 days. (e) Relative mRNA expression levels of *ACSL3* in cells after induced differentiation for 0, 2, 4, 6 and 8 days. Results are presented as means ± SEM; *n* = 3; Labels with different superscripts indicate extremely significantly different values (*P* < 0.01).

To examine the functional impact of *ACSL3* on adipocyte differentiation, we established a model for porcine intramuscular preadipocyte differentiation. The morphology of primary intramuscular preadipocytes after cultivation is shown in Figure 4(a). Oil Red O staining revealed the generation and morphology of lipids in the cultured intramuscular adipocytes 4 days after induction (Figure 4(b)). After preadipocyte differentiation, the expression levels of the lipogenesis associated genes *PPAR-γ* and *SREBP-1c* increased rapidly and peaked on the second day (Figure 4(c)), while the steatolysis associated genes *ATGL* and *SIRT1* peaked on the fourth day (Figure 4(d)). To demonstrate the role of *ACSL3* in porcine intramuscular adipocyte differentiation, we measured the expression of *ACSL3* during preadipocyte differentiation. *ACSL3* mRNA expression peaked on the second day and then decreased gradually, as shown in Figure 4(e).

**Figure 5.**
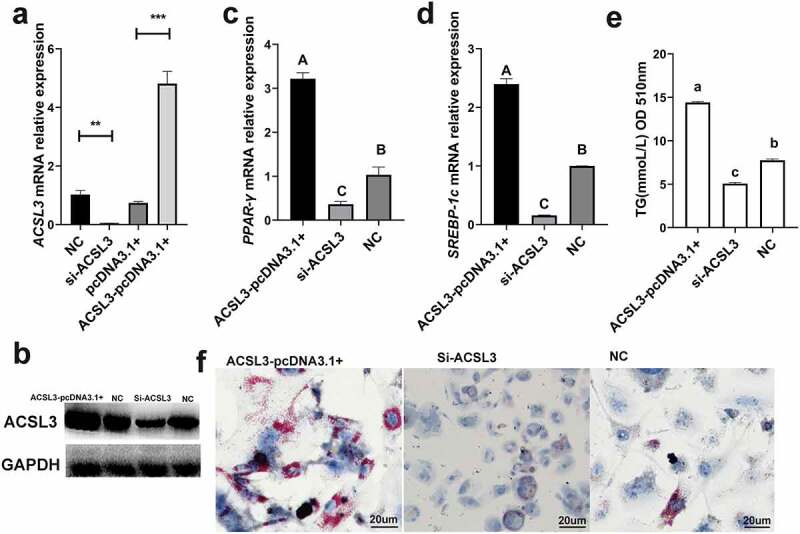
*ACSL3* promotes the lipid droplet accumulation. (a) Relative mRNA expression levels of *ACSL3* in cells transfected with Si-ACSL3, ACSL3-pcDNA3.1+ and pcDNA3.1+ and blank negative control (NC). (b) Western blots showing ACSL3 levels of cells transfected with Si-ACSL3, ACSL3-pcDNA3.1+ and NC, *GAPDH* as the internal reference. (c) Relative mRNA expression levels of *PPAR-γ*. (d) Relative mRNA expression levels of *SREBP-1c*. (f) Oil Red O staining of terminally differentiated adipocytes. Results are presented as means ± SEM; n = 3; ** *P* < 0.01; *** *P* < 0.001; Labels (a, b, c) indicate significantly different values (*P* < 0.05). Labels (A vs. B) indicate extremely significantly different values (*P* < 0.01).

The Si-ACSL3 oligonucleotides were designed to knock down *ACSL3*, and the ACSL3-pcDNA3.1+ was constructed to simulate the overexpression of *ACSL3*. q-PCR and Western blotting were used to calculate the interference and overexpression efficiencies (Figure 5(a-b)). The mRNA levels of *PPAR-γ* (Figure 5(c)) and *SREBP-1c* (Figure 5(d)) were significantly reduced during the knockdown of *ACSL3*, and the TG content of these cells was also markedly reduced as determined at OD510 (Figure 5(e)) and by Oil Red O staining (Figure 5(f)). By contrast, when the cells were transfected with ACSL3-pcDNA3.1+, the mRNA levels of *PPAR-γ* (Figure 5(c)) and S*REBP-1c* (Figure 5(d)) were significantly increased, the quantification of TG (Figure 5(e)) and Oil Red O staining (Figure 5(f)) revealed that overexpression of *ACSL3* significantly increased the accumulation of lipid droplets. These results demonstrate that *ACSL3* positively affects porcine adipocyte differentiation.

### 2.5 miR-26a-5p inhibits porcine adipogenesis differentiation by targeting ACSL3

**Figure 6.**
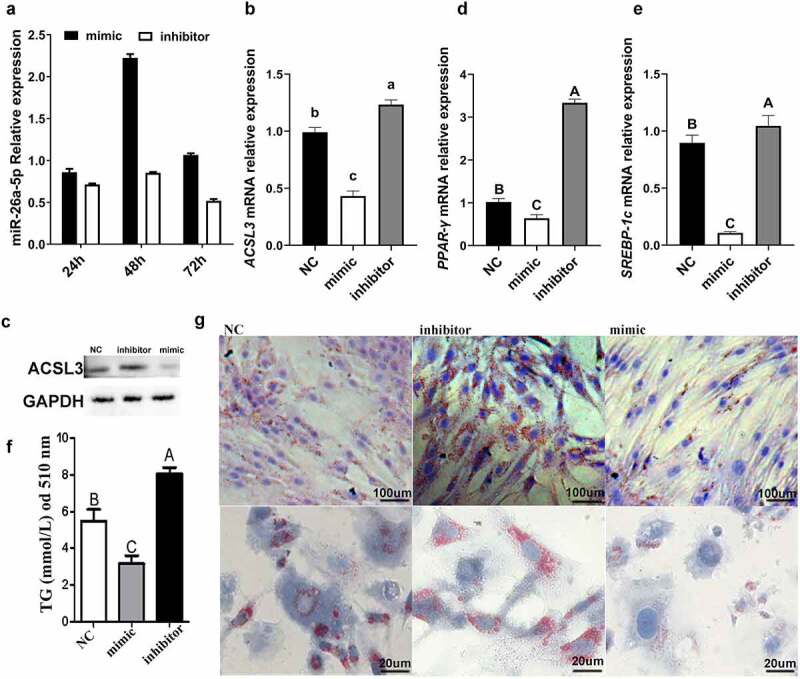
miR-26a-5p inhibits lipogenesis in porcine preadipocytes. (a) The miR-26a-5p levels in cell transfected with mimic/inhibitor on 24 h, 48 h, and 72 h. (b) Relative mRNA expression levels of *ACSL3* in cells transfected with miR-26a-5p mimic, inhibitor and NC. (c) Western blotting showing proteins from cells transfected with miR-26a-5p mimic, inhibitor and NC (*GAPDH* as the internal reference). (d) Relative mRNA expression levels of *PPAR-γ* in cells transfected with miR-26a-5p mimic, inhibitor and NC. (e) Relative mRNA expression levels of *SREBP-1c* in cells transfected with miR-26a-5p mimic, inhibitor and NC. (f) TG levels in the miR-26a-5p mimic, inhibitor and NC. (g) Oil Red O staining of terminally differentiated adipocytes. Results are presented as means ± SEM; n = 3; Labels (a, b, c) indicate significantly different values (*P* < 0.05). Labels (A vs. B) indicate extremely significantly different values (*P* < 0.01).

To test if miR-26a-5p affects adipocyte differentiation, a mimic and an inhibitor of miR-26a-5p were transfected into porcine intramuscular preadipocytes. After 48 h of transfection, miR-26a-5p had the highest expression levels in the mimic group (Figure 6(a)). The miR-26a-5p levels in the inhibitor group stayed relatively consistent throughout the experiment (Figure 6(a)). The expression of *ACSL3* was down-regulated by miR-26a-5p (Figure 6(b-c)), and miR-26a-5p mimic significantly suppressed expression of the lipogenesis genes *PPAR-γ* and *SREBP-1c* (Figure 6(d-e)). miR-26a-5p mimic resulted in decreased lipid droplet accumulation, whereas the miR-26a-5p inhibitor resulted in an increased lipid droplet accumulation, as measured by TG (Figure 6(f)) and Oil Red O staining (Figure 6(g)).

## 3. Discussion

MicroRNAs silence mRNAs and regulate gene expression by binding target sequences by complementary base-pairing, resulting in post-transcriptionally degraded target gene expression [25]. Since microRNAs were reported, they have played important roles in many biological processes, and people are more concerned about their role in regulating cell differentiation [26]. Current miR-26a-5p research largely focuses on tumorigenesis, while little is known about the role of miR-26a-5p in the regulation of porcine preadipocyte differentiation.

Bioinformatics analysis suggested that *ACSL1, ACSL3* and *ACSL4* were potential miR-26a-5p targets, which participate in the adipocytokine signalling pathway. ACSLs are involved in nearly all metabolic pathways in mammals, including complex lipid synthesis, protein modification, and β-oxidation [27]. Five ACSL members can activate and channel various fatty acids to different metabolic fates [28]. The expression of *ACSL3* and *ACSL4* in the high-fat group and low-fat group reached a significant level, indicating that they may play more important roles in the process of porcine lipid synthesis.

At present, there are few studies of *ACSL3* in pigs. Among ACSL family members, ACSL3 is the most highly expressed during the process of milk fat synthesis in sows, and the expression level of *ACSL3* mRNA increases significantly with milk production [29]. According to the previous studies, *ACSL3* can regulate the activity of transcription factors in adipocyte differentiation to maintain lipid balance [30] and is involved in the production of 3T3-L1 adipocytes in mice [31]. In this study, we demonstrate that*ACSL3* plays a more important role in porcine adipocyte differentiation. We show that *ACSL3* mRNA is expressed throughout the entire differentiation process of porcine intramuscular preadipocytes and abundant in the early term after adipocyte differentiation. The results of overexpression and knockdown showed that ACSL3 promotes the accumulation of lipid droplets in intramuscular preadipocytes.

Furthermore, we demonstrate that miR-26a-5p binds the seed sequence of the 3ʹUTR region of the *ACSL3* gene. Meanwhile, miR-26a-5p inhibits the accumulation of lipid droplets in intramuscular preadipocytes. Although we have verified that the expression of *ACSL3* is regulated by miR-26a-5p, neutralizing *ACSL3* blocks the effect of miR-26a-5p should be researched in the future.

In summary, this study demonstrated that *ACSL3* promotes the lipid synthesis of porcine preadipocytes and that *ACSL3* is a bona fide target of miR-26a-5p. Furthermore, miR-26a-5p is a repressor of porcine preadipocyte differentiation and plays a vital role in *ACSL3*-mediated adipogenesis. Our study suggests that miR-26a-5p and *ACSL3* may be promising biomarkers for intramuscular fat.

## 4. Materials and methods

### 4.1 miRNA target gene prediction and functional analyses

The miRPathv3 (http://snf-515788.vm.okeanos.grnet.gr/) contains data from microT-CDS (http://www.microrna.gr/microT-CDS), TarBase (http://www.microrna.gr/tarbase) and TargetScan (http://www.targetscan.org/vert_72/), which can more accurately predict the targeting genes of microRNAs. microRNA targets were predicted using miRPathv3, and overlapping target genes were performed with GO term and KEGG enrichment analyses. Figures were prepared using the R package ‘ggplot2’ [32]. The free energy of the miR-26a-5p and *ACSL3* interaction was calculated using RNAhybrid 2.2 (http://bibiserv.techfak.uni-bielefeld.de/rnahybrid/) [33].

### 4.2 Quantitative real-time PCR

Among the 63 samples previously collected in our laboratory, we defined the pigs with the top 10% of intramuscular fat content as the high group (H-group, IMF 3.3–6%) and the pigs with the last 10% of intramuscular fat content as the low group (L-group, IMF less than 2.2%). Three samples were randomly selected from the high and low groups individually for this study. All tissues (heart, liver, spleen, lung, kidney, and longissimus dorsi) were collected under sterile conditions. Immediately after excision, tissues were flash-frozen in liquid nitrogen and then stored at −80 ^ο^C until RNA extraction. Total RNA extraction was done using miRcute miRNA isolation kit (TianGen, China).

The cDNA and miRNA-specific cDNA syntheses were performed using the PrimeScript RT reagent kit with gDNA Eraser (TaKaRa, China) and the PrimeScript RT reagent kit (TaKaRa, China) separately, following manufacturer’s instruction. qRT-PCR were run on a Roche LightCycler96 (Switzerland), using TB GREEN Premix Ex Taq II (Tli RNaseH PLUS) (TaRaKa, China), or TB GREEN Premix Ex Taq GC (Perfect Real Time) (TaRaKa, China). The 2^−ΔΔCt^ method [34] was used to quantify the relative expression level of the transcript mRNA or miR-26a-5p. U6 and glyceraldehyde phosphate dehydrogenase (GAPDH) were the housekeeping genes for miRNA and mRNA, respectively. Sequences for all primers used in this study are shown in supplementary Table S2.

### 4.3 Luciferase assay

The binding site of miR-26a-5p and 3ʹUTR of *ACSL3* was predicted using TargetScan. The wild-type *ACSL3* in this region was amplified by PCR using PrimeSTAR® HS DNA Polymerase (TAKARA, China) and purified by TIANgel Midi Purification Kit (TIANGEN, China). The product was digested by restriction endonucleases Sac I and Sal I (TransGen, China), and the product was linked to the pmirGLO Dual Luciferase Reporter vector by DNA ligation kit (TAKARA, China), and the constructs were validated by sequencing. The wild-type *ACSL3* plasmid was named WT. The mutant-type *ACSL3* plasmid was mutated on the WT by using the Fast Mutagenesis System (TransGen, China), mutant primers were shown in Table S3. The mutant-type *ACSL3* plasmid is termed MUT. Using Lipofectamine 2000, the WT and MUT were co-transfected with miR-26a-5p mimic (sense: 5’-UUCAAGUAAUCCAGGAUAGGCU-3’; antisense: 5’-CCUAUCCUGGAUUACUUGAAUU-3’) or miR-26a-5p inhibitor (sense: 5’-AGCCUAUCCUGGAUUACUUGAA-3’) into PK-15 cells. We tested luciferase activity according to the specification of the Dual Luciferase Reporter Assay System (Promega, USA) after 48 hours of transfection.

### 4.4 Cell culture and adipocyte differentiation

We established a porcine preadipocyte differentiation model, following the previous study [8]. Eight 5-day-old Yorkshire pigs were purchased from the experimental farm at the Chinese Academy of Agricultural Sciences. After euthanasia, the backs of the pigs were sterilized with 75% alcohol and detergent, the tissue was separated from the longest muscle position of the back and placed into sterile PBS with 300 U/mL penicillin-streptomycin, and then washed three times with the same solution. The muscle tissue was then cut into pieces approximately 25 cm^3^. After 2 hours of digestion with 1 mg/mL of type II collagenase (Solarbio, China) at 37°C, the primary IMF cells were harvested and incubated in DMEM/F12 (Gibco, USA) supplemented with 10% foetal bovine serum (FBS, BI, Israel) and 100 U/mL penicillin–streptomycin (Solarbio, China) at 37°C in a humidified 5% CO_2_ atmosphere. After 2 hours, the cells were washed twice with PBS and then suspended in fresh culture media; incubation continued until the cell density reached 90%, at which point they were passaged.

Cell differentiation was induced by incubating confluent cells (in 6-well plates) for 2 days in a differentiation medium (DMEM/F12 supplemented with 10% FBS, 0.5 mM 3-isobutyl-1-methylxanthine (IBMX), 1 μM dexamethasone (DEX), and 5 μg/mL of insulin). After 2 days, this medium was changed to DMEM/F12 with 10% FBS and 5 μg/mL of insulin.

### 4.5 Overexpression and knockdown assay of ACSL3

The whole coding region of *ACSL3* was amplified by the Primestar polymerase (Takara, China) with a forward primer containing the endonuclease Kpn I sequence and a reverse primer containing the endonuclease Xho I sequence. The PCR fragments were restriction enzyme digested and ligated into pcDNA3.1(+) expression vectors with Solution I (Takara, China). ACSL3-pcDNA3.1(+) were verified by bidirectional sequencing and transformed into E. coli DH5α competent cells for overexpression, then purified using an Endo Free Plasmid Purification Kit (TIANGEN, China).

According to the porcine *ACSL3* mRNA sequence (GenBank number NM_001143698.1) on GenBank as the template, we designed a Si-ACL3 (sense: 5’-GCGGUGAUCAUGUAUACAATT-3’; antisense: 5’-UUGUAUACAUGAUCACCGCTT-3’).

### 4.6 Transfection

The porcine preadipocyte cells were detached from the culture plates with 0.25% trypsin (Gibco, USA) and transferred to six-well plates with antibiotic-free medium. Using Lipofectamine 2000 (Invitrogen, Carlsbad, CA), cells were transfected with 50 nM of miR-26a-5p mimic, 100 nM of miR-26a-5p inhibitor. Si-RNAs were diluted to 20 μM and transfected into IMF cells as above. The total RNA and protein were isolated from the transfected cells 48 h post-transfection.

### 4.7 Western blotting

After 48 hours of transfection, the cells were washed twice with PBS and lysed with 1% PMSF (Beyotime, China) RIPA Lysis Buffer (Beyotime, China). The mixed solution obtained in the previous step was centrifuged at 4°C for 5 minutes at 12,000 rpm. After centrifugation, the supernatant was collected which was a protein solution. The concentration of protein from IMF cell samples was determined using a BCA assay kit (TIANGEN, China). The sample volume per hole was 20 μg protein, separated by 6% 12 alkyl sulphate polyacrylamide gel electrophoresis, then transferred to PVDF membrane (Millipore, Billerica, MA). The PVDF membrane was sealed with 10× TBST (Beyotime, China) containing 5% skimmed milk powder for 2 h at 37°C and then cleaned with 1× TBST for 3 times. Depending on the marker size, cut the PVDF membrane to the proper size. The treated membranes are incubated separately with rabbit primary antibodies (1:1000), anti-ACSL3 (1:5000) and anti-GAPDH (1:5000) (ABclonal, China) for 24 h at 4°C, followed by incubation with secondary antibodies (1:1000) (Beyotime, China) for 2 h at 37°C. Proteins were visualized using chemiluminescent peroxidase substrate (Beyotime, China).

### 4.8 Oil red O staining and quantification of triglycerides

The porcine preadipocyte cells in six-well plates, which had been induced by differentiation medium, the preadipocyte cells were washed with PBS three times. The cells were covered with 1 mL of 4% formaldehyde per well at room temperature. After fixation for 20 minutes, PBS washed the six-well plates three times. The original oil red O solution (Solarbio, China) was added with 3:2 water to make oil red staining solution. The cells were covered with oil red staining solution for 20 minutes at room temperature and washed with PBS. The six-well plates were stained with haematoxylin (Solarbio, China) for 1 min, washed with ice water, and then observed using an OPTIKA XDS-3 microscope (Italy).

The porcine preadipocyte cells in six-well plates, which had been induced by differentiation medium, the cells were treated with 0.25% trypsin until separation, centrifuged for 3 min at 1000 rpm, and then TG content in cell homogenate was determined according to the instructions of TG assay kit (Nanjing Jiancheng Bioengineering Institute, China).

### 4.9 Statistical analysis

All the experiments were carried out in triplicate, and the data were expressed as mean ± Standard Error of Mean (SEM). The two groups were compared by *t*-test, and the multiple groups were compared by one-way ANOVA. Multiple comparisons were made using the Dunnett’s test. *P* < 0.05 was considered significant. SPSS version 11.0 software (SPSS, Chicago, IL) was used for all statistical analysis.

## Supplementary Material

Supplemental Material

## Data Availability

All data generated or analysed during this study are included in this published article and its supplementary files.

## Abbreviations

IMF, intramuscular fat; ACSL, long-chain acyl-CoA synthetase; ACSL3, acyl-CoA synthetase long-chain family member 3; GO, Gene Ontology; KEGG, Kyoto Encyclopaedia of Genes and Genomes; CoA, coenzyme-A.
